# Perception and Knowledge of Patients from Different Regions in the Kingdom of Saudi Arabia towards Oral Hygiene and Oral Hygiene Aids

**DOI:** 10.3390/healthcare9050592

**Published:** 2021-05-17

**Authors:** Shamoukh Alshahrani, Abrar Alshuaibi, Malak Alkhaldi, Pradeep Koppolu

**Affiliations:** Department of Preventive Dental Sciences, College of Dentistry, Dar Al Uloom University, Riyadh 13314, Saudi Arabia; shomo52525@gmail.com (S.A.); abraralshuaibi@gmail.com (A.A.); malakalkhaldi2020@gmail.com (M.A.)

**Keywords:** oral hygiene aids, dental floss, tongue brushing

## Abstract

Aim: The present study aims to evaluate the perception and awareness of interdental aids in different regions of Saudi Arabia. Methods: A cross-sectional questionnaire-based study was conducted, in order to evaluate the perception and knowledge of patients towards oral hygiene products among the population of the Kingdom of Saudi Arabia. In total, 812 out of 1124 participants responded and completed the survey (response rate 72.2%). The data were collected using a self-administered structured questionnaire in English and Arabic. All statistical analyses were carried out using the SPSS 20 software. *p* < 0.05 was used to indicate statistical significance. Results: A total of 812 participants responded, of which 486 participants (60%) declared using a toothpaste and toothbrush for cleaning their teeth. The cohort consisted of 274 (34%) females who brushed twice daily, while 96 (33%) males brushed their teeth once a day and 18% of the participants did not even brush once a day. The results indicated that 332 (64%) female participants and 174 (60%) male participants had perception and knowledge of the use of dental floss or any other device to clean between their teeth, while 174 (48.50%) male participants and 174 (49.10%) female participants cleaned their tongue with the same brush, rather than using a tongue scrubber or any other aids. Conclusion: The total awareness of interdental aids in Saudi Arabia is unsatisfactory, as demonstrated by the participants not being conscious or informed about the maintenance of their oral health. A majority of participants did not report adopting basic techniques, such as tongue brushing. This study reveals that no interdental aids were used by 16% of the participants. Thus, it is crucial to develop an effective educational program which emphasizes oral healthcare in this population.

## 1. Introduction

Oral health is now recognized to be equally important to general health. The oral cavity is the “mirror” of general health [1]; however, oral and general health status relies on the complex interplay of many variables, including the personal awareness, attributes, behaviors, and perceptions of the individual. Awareness of oral health is an essential requirement for health-related behavior, and awareness increases with age. Health-related behaviors are defined as the activities carried out by individuals to protect, encourage, or preserve health and to prevent disease [2]. Oral care products are those with the purpose of mouth cleansing, breath freshening, and maintaining oral hygiene. As the dental industry has been continuously expanding, a variety of oral care products are available within the market, making the selection process rather challenging. Several approaches could have an impact on the selection of oral care products, as these play a critical role in improving oral health and preventing dental diseases [3]. The most-used oral hygiene devices include toothbrushes and toothpaste. Other oral hygiene aids are also used by individuals, either on the recommendation of a dentist or voluntarily. Factors such as education, wages, advertisements, and personal choices involving taste/flavor, color, and presentation of the product, among others, play roles in the choice of an oral hygiene product [2]. The maintenance of adequate oral health depends on the adoption of specific behaviors, including regular dental check-ups [4], brushing frequency [5], diet and sugar consumption [6], dental floss use, and other methods of interproximal cleaning [7]. These approaches play a critical role in the prevention of decay and periodontitis [8]. Additionally, awareness of oral health is essential for the cultivation of beneficial habits regarding dental hygiene. Various studies have established a strong correlation between oral health status and the level of awareness. As healthcare practitioners primarily seek to promote health and give prevention information, they should set high standards of oral health expertise which comply with professional guidelines [9]. Health education is the transfer of knowledge and aptitude required for enhancing quality-of-life, as it is a commonly recognized solution for avoiding diseases. Furthermore, the purpose of the proposed health promotion program is not only to develop new habits, but also to sustain and enhance healthy behaviors that can promote health in individuals and communities [10]. Therefore, the present study was conducted to assess the perception and knowledge of patients from different regions in the Kingdom of Saudi Arabia regarding oral hygiene products.

## 2. Materials and Methods

A cross-sectional questionnaire-based study was conducted for 2 months (August–September 2020), in order to understand the perception and knowledge of patients towards oral hygiene products among the population of the Kingdom of Saudi Arabia. An electronic copy of the questionnaire was prepared using Google forms (Alphabet Inc., Mountain View, CA, USA). A short electronic link was then created and distributed through social media. A pilot study was conducted to check the validity and comprehensibility of one questionnaire, which included all the questions and was circulated to 80 participants, and its results yielded an acceptable form with minor changes based on the responses. Considering the expected proportion of 65.7% from the pilot study, with the relative precision of 5% and desired confidence level of 95%, the calculated sample size came up to 802, which was rounded off to 810. The formula for calculating the sample size is as follows:

*n* = Z^2^_1- α/2_ × (1-P)/ε^2^ × P (1)

where P = Expected proportion, _1- α/2_ = Desired confidence level, ε = relative precision.

In total, 812 out of 1124 participants responded and completed the survey (response rate 72.2%). The data were collected using a self-administered structured questionnaire in English and Arabic. The questionnaire contained 25 multiple-choice questions related to the oral hygiene method used to clean the teeth, the type and brand of dentifrice used, frequency of brushing, duration of tooth brushing, type of toothbrush, and the reason for choosing a specific brand of dentifrice. The aim of the study was presented first to the participants, and they were encouraged to provide their consent and participate by clicking on the attached link to complete the survey items. The confidentiality and anonymity of the gathered information were emphasized. A total of 812 participants responded to the online questionnaire. The collected data were entered in a database, then processed and analyzed using Excel (Microsoft Excel, Version 2020) (Microsoft, Redmond, WA, USA), while the statistical analysis was performed using the Statistical Package for the Social Sciences version 20 (SPSS 20) (IBM, Armonk, NY, USA) software. A quality control check of the data entries was conducted before the data analysis. Depending on the nature of the variables—for example, for demographic- and profession-related data—we used descriptive analysis (percentage and frequency). To find the association between categorical variables, the chi-square test was used (with statistical significance set at 0.05).

## 3. Results

**Figure 1:**
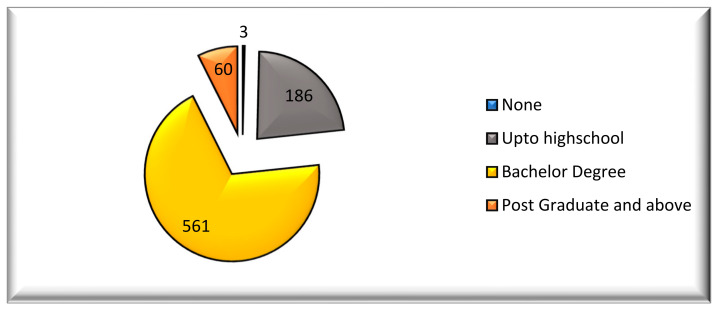
Distribution of education levels.

**Figure 2:**
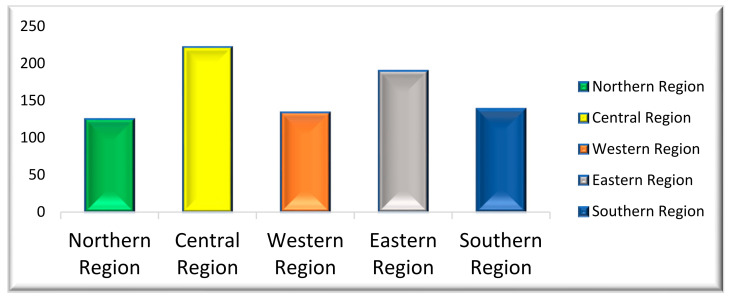
Distribution of regions in Saudi Arabia.

The cohort of 812 subjects consisted of 519 (64.9%) females and 291 (35.9%) males (see Table 1). A total of 449 (55%) of the participants belonged to the 20–30 age group (Table 1). The distribution of the study population, according to education level, showed that 0.4% (3) had no educational level, 23% (186) had up to high school, 69.3% (561) had a bachelor’s degree, and 7.4% (60) were postgraduate and above (Table 1 and Figure 1). Based on the geographical distribution, about 15.4% (125) of the population were from the northern region, 27.4% (222) were from the central region, 16.5% (134) were from the western region, 23.5% (190) were from the eastern region, and 17.2% (139) were from the southern region (Table 1 and Figure 2). Most participants (60%) used a toothpaste and toothbrush for cleaning their teeth, compared to toothpaste, toothbrush, and floss (or miswak) (Table 2). Around 91.2% of respondents from the northern region, 98.2% of those from the central region, 97% of those from the western region, 95.8% of those from the eastern region, and 91.2% of those from the southern region had a perception and knowledge about cleaning their teeth, where the difference was statistically significant (*p* = 0.028). Among the participants, 34% of females brushed twice daily, while 33% of males brushed their teeth once a day (Table 3). Interestingly, most of the individuals usually brushed their teeth for about 1 min (Table 3). A total of 98.1% of females and 92.4% of males had a perception about cleaning their teeth, where the difference was statistically significant (*p* = 0.001). Among the different educational levels of the subjects, 41% of bachelor degree subjects used a medium brush, whereas 33% of the illiterates, 37% of the up to high school, and 40% of postgraduates and above used a soft brush (Table 2). Among the different educational levels of the study participants, 100% of the no education level respondents (illiterates), 92.5% of up to high school, 97.5% of bachelor degree, and 93.3% of postgraduate and above had a perception about cleaning their teeth, where the difference was statistically significant (*p* = 0.014). Interestingly, 358 (34.1%) of the participants used a horizontal brushing technique, while 316 (30.1%) of the participants used a vertical brushing technique. The usual recommended technique of brushing is vertical as it leads to less abrasion compared to the horizontal technique. Among all parameters and sub-parameters, all sex, age group, region, and education level subjects changed their brush every 3 months of duration, where the parameters of sex and age group displayed statistically significant differences (*p* < 0.05). Almost 45.1% (365) of the study participants changed their brush every 3 months (Table 3). Ideally the brush needs to be replaced every 3 to 4 months.

Among all parameters and sub-parameters, around 35% of males and 41% of females used a soft brush, and the difference is statistically significant (*p* = 0.001). Furthermore, 47% of the population in the 31–40 age group used a medium brush, while about 38% in the 20–30 age group, 39% of the 41–50 age group, and 47% of the population in the >50 age group used a soft brush, and the difference is statistically significant (*p* = 0.001). (Table 4). The brush types which are usually advised to brush with are soft and ultrasoft.

The responses of the participants regarding their knowledge of interdental aids are summarized in Table 5. We deduced that 84% (680) knew about interdental aids, while 16% (130) did not possess the relevant knowledge. Furthermore, 55.4% of participants in the 20–30 age group, 19.1% in the 31–40 age group, 17.3% in the 41–50 age group, and 8.1% in the > 50 age group indicated having relevant knowledge. The data indicated that, among the different regions of Saudi Arabia, approximately 76.80% of the population in the northern region, 82.90% in the central region, 85.80% in the western region, 87.90% in the eastern region, and 84% in the southern region answered that they had some knowledge about interdental aids. Among the different educational levels, 81.20% of up to high school, 84.80% of bachelor’s degree, and 83.30% of postgraduate and above participants were also aware of interdental materials (Table 5). About 64% (334) of females and 59% (173) of males used interdental aids; 40% (118) of males commonly used a toothpick as an interdental aid, while 41% (217) of females used dental floss (Table 6). Additionally, 50% of females and 45% of males used mouthwash, with no statistically significant difference being noted (*P* > 0.05). Moreover, 51% of the 20–30 age group and 61% of the above 50 age group in the study population used mouthwash; on the other hand, only 43% of the 30–40 age group and 41% of the 41–50 age group used mouthwash (Table 6). Meanwhile, 48% of males and 49% of female chose to clean their tongue with the same brush, instead of using a tongue scrubber or any other aids (Table 6). Almost 79% of males and 69% of females did not wear braces, bridges, or any fixed prosthesis (Table 6), while 55% of males and 56% of females agreed that their dentists did not explain how to properly brush their teeth or how to use interdental aids (Table 6).

## 4. Discussion

In the current study, more than 60% of respondents used toothpaste and a toothbrush for cleaning their teeth, compared to toothpaste and toothbrush with floss, miswak, no brushing, or other products. A previous study by Hussain et al. (2018) found that most participants (88%) used a toothbrush to clean their teeth, 5.5% used toothpowder, 5% used their finger, and 1.5% did not use any method for cleaning their teeth [11], which was consistent with the study by Al-Qahtani et al. (2020). Furthermore, 79.4% of schoolchildren used a toothbrush and toothpaste, whereas 17.8% used miswak for cleaning their teeth [12]. A similar study, by Almulhim (2016) in Riyadh City, showed that 82% of respondents used a toothbrush and 4% used miswak, while 3% used dental floss [13]. An alternative study has been conducted, by Elsabagh et al. (2018), on oral hygiene knowledge, attitude, practice, and self-perception of personal dental appearance among Majma’ah University female students in the Kingdom of Saudi Arabia. They found that only 36% of students used a brush and dental floss for tooth cleaning [14]. Conversely, the results of the study by Hammadi et al. (2020) in the southern region evaluated the types of oral hygiene aids used by participants; these included toothbrush (46.5%), miswak (8%), and both toothbrush and miswak (44.7%). A similar study carried out in Jeddah City revealed that 84% of participants used a toothbrush, 40% used miswak, and 20% used dental floss [15]. According to the study of Almassri et al. (2019), 971 (55%) participants used a toothbrush and toothpaste to clean their teeth, while 234 (23%) used mouthwash, and 177 (17%) used miswak [16]. In the current study, 33% of males brushed their teeth once a day and 34% of females brushed their teeth twice a day, while the study by Hussain et al. (2018) revealed that 65% of the participants brushed only once daily, 27.5% brushed twice daily, 3.5% brushed occasionally, and only 4% brushed more than twice daily [11]. Another study by Elsabagh et al. (2018), on oral hygiene knowledge, attitude, practice, and self-perception of personal dental appearance among Majma’ah University female students, showed that 42.4% brushed twice a day, with 29% brushing for 2 min. This finding is similar to that in India, where 50.4% of respondents have been reported to brush their teeth twice a day—which is the international endorsement of brushing—and 83.9% did not use dental floss [14]. Another study by Al-Qahtani et al. (2020) found that 33.1% of schoolchildren brushed their teeth daily; among these, 50.2% brushed their teeth once daily, whereas 35.8% brushed their teeth twice daily [12]. The study by Al-Hammadi et al. (2020) reported that 28.2% brushed once daily, 37.6% brushed twice daily, 28.4% brushed whenever required, and 5.9% brushed infrequently. In the present study, the prevalence of daily brushing was similar to that reported in the study conducted to assess the level and aspects of knowledge, attitude, and practices related to oral health among pilgrims visiting Madinah: 21.2% participants brushed once a day, 30.7% brushed twice a day, and 8.4% never brushed [15]. In a study by Almassri et al. (2019), about 381 (39%) patients cleaned their teeth twice a day, followed by 329 (33%) subjects who cleaned their teeth once a day [16]. Al-Hammadi et al. (2020) showed that 84.7% of participants preferred to continue using miswak in combination with other teeth cleaning methods that were beneficial [15], in agreement with the finding of the study conducted by Darout et al. (‎2016) in the Jazan region in Saudi Arabia. This study concluded that the miswak stick was equally used as a toothbrush for oral hygiene among secondary school students [17]. In our study, the majority of the study subjects from all regions used a medium brush, except for those in the southern region, who tended to use a soft brush. According to Hussain et al. (2018), 45% of respondents used a medium brush and 35% used a soft brush [11]. The study by Qahtani et al. (2020) demonstrated that, among those who used a toothbrush, about 54.8% did not know the type they used, while only 30.1% of the schoolchildren in the study used a soft brush [12]. A similar study by Almulhim (2016) revealed that 27.35% of fathers and 37.35% of mothers reported that the soft type was the most commonly used brush, while 0.66% of mothers answered that the most commonly used was the hard brush, showing a highly statistically significant difference between fathers and mothers (*p* < 0.008) [13]. According to sex, age group, region, and education level, the subjects predominantly used a horizontal brushing technique, except for the postgraduate and above education level and northern region people of Saudi Arabia, who used a vertical brushing technique. A study by Darout et al. (2016) in the Jizan region, Saudi Arabia, found that less than half of both males and females who used a toothbrush applied both horizontal and vertical methods [17]. On the other hand, Almassri et al. (2019) reported that 570 (46%) of patients used the toothbrush in a circular motion while brushing their teeth [16]. The study by Graça et al. (2019) showed that, although fluoride toothpaste was widely used by participants (814; 93.2%), some difference was noted in Romania, where this type of toothpaste was not used as much as in the other countries (37; 8.1%). Interestingly, whitening and desensitizing toothpastes seemed to be popular among adolescents in Portugal and Romania (*p* < 0.001) [18]. Agrawal et al. (2020) found that the majority of individuals (39.3%) chose their toothpaste based on cost, followed by the taste of the toothpaste (34%) [19]. In the current study, we found that the subjects changed their brush every 3 months. Graça et al. (2019) showed that Portuguese participants changed their toothbrush less often than their international counterparts (*p* < 0.001). [18] In this study, a total of 64% of females and 60% of males displayed a perception and knowledge on the use of dental floss or any other device to clean between the teeth. Conversely, in the study by Kamil et al. (2017), in Jizan, Saudi Arabia, among 15–34-year-old individuals, only 323 (21.5%) used interdental cleaning aids; of these, only 50 used these aids regularly, while the others used them intermittently. A total of 854 (53.5%) subjects chewed miswak, of which 20 chewed only miswak, while the majority (*n* = 834) practiced toothbrushing in addition to miswak [20]. Chakraborthy et al. (2017) demonstrated that only a few subjects used dental floss for interdental cleaning [21], similar to the findings of a study among participants visiting a dental college in India (Hussain et al., 2018) [11]. In our study, 48.50% of males and 49.10% of females cleaned their tongue with the same brush without using any tongue scrubber or other aids. The study by Hussain et al. (2018) showed a lack of awareness among participants about basic oral health maintenance techniques, such as tongue cleaning [11]. In a study by Kamil et al. (2017), when students were asked if they knew any ideal aid that could clean dental plaque from interdental spaces, only 25.8% answered positively. In the present study, 21.2% of the participants had heard about dental floss, and 4.3% knew that dental floss is essential for removing plaque and debris from the interdental area. However, only one participant had seen a person using dental floss, while 1.8% knew that the use of dental floss should be used customarily and daily along with tooth brushing, as the remaining participants used it to remove interdental food debris. Furthermore, 75.3% of the participants in the current study believed that dental floss harms the interdental gingiva; this proportion was higher than that (24.3%) reported by Shazia et al. (2016) [20,22]. Compared to the study conducted in Portugal, Romania, and Sweden (Graça et al., 2019), and that carried out among adult population in Saudi Arabia (Almassri et al., 2019), more than half of the participants (469; 54%) had never used dental floss. A maximum of 406 (35%) subjects used dental floss as a secondary method for plaque control, while 306 patients (25%) had used toothpicks [16,18], which was lower than the figure in the current study, wherein 41% of males commonly used a toothpick to clean the interdental area and 42% of females used dental floss. Moreover, 21% and 30.30% of females wore a fixed partial denture (FPD). In the study by Harini et al. (2019), 40% of the patients were females and 60% were males, where 65% of the patients had a FPD. In our study, 20.60% of males and 27.60% of females used special cleaning aids to clean their prosthesis, which was higher than that (10%) in patients in the study by Harini et al. (2019) who used such cleaning aids. Furthermore, 50% of the patients used chlorhexidine, 40% used floss, and 10% used an interdental brush to clean their prosthesis [23].

## 5. Conclusions

In conclusion, the oral hygiene practices are an ignored area of care among some of the study population. Many of the participants were not conscious and informed about oral health maintenance and interdental aids. A total of 51% of the participants are not using mouthwash as a routine oral hygiene practice. Even though 68% of the participants regularly performed tongue cleaning, there is a need to educate the rest of the people (32%) regarding the importance of tongue brushing. A total of 18% of the participants do not brush even once in a day, and 16% of the participants are not aware of interdental aids. Since plaque removal is essential to reduce gingival inflammation, a need for developing an educational program is necessary. The practitioners should emphasize the oral hygiene instructions and keep patients motivated regarding preventive measures which is imperative for this population.

## Figures and Tables

**Table 1:** Distribution of study population, according to gender, age, region, and education level.

Parameters	Sub-Parameters	Frequency	Percent
Sex	Male	291	35.9
	Female	519	64.1
Age Group	20–30	449	55.4
	31–40	155	19.1
	41–50	140	17.3
	50 and Above	66	8.1
Region	Northern Region	125	15.4
	Central Region	222	27.4
	Western Region	134	16.5
	Eastern Region	190	23.5
	Southern Region	139	17.2
Education level	None	3	0.4
	Up to High School	186	23
	Bachelor’s Degree	561	69.3
	Postgraduate and Above	60	7.4

**Table 2:** Perception and knowledge of different parameters on “What do you use to clean your teeth?”.

Variables			Toothbrush and Toothpaste	Toothbrush, Toothpaste, and Floss	Miswak	I Don’t Brush My Teeth	Other	*p* Value
Sex	Male	N	189	61	19	12	10	0.001 *
		%	64.90%	21.00%	6.50%	4.10%	3.40%	
	Female	N	312	184	9	5	9	
		%	60.10%	35.50%	1.70%	1.00%	1.70%	
Age Group	20–30	N	267	155	5	10	12	0.001 *
		%	59.50%	34.50%	1.10%	2.20%	2.70%	
	31–40	N	103	45	3	4	0	
		%	66.50%	29.00%	1.90%	2.60%	0.00%	
	41–50	N	89	34	11	2	4	
		%	63.60%	24.30%	7.90%	1.40%	2.90%	
	50 and above	N	42	11	9	1	3	
		%	63.60%	16.70%	13.60%	1.50%	4.50%	
Region	Northern Region	N	83	27	9	5	1	0.001 *
		%	66.40%	21.60%	7.20%	4.00%	0.80%	
	Central Region	N	140	75	4	1	2	
		%	63.10%	33.80%	1.80%	0.50%	0.90%	
	Western Region	N	76	42	5	2	9	
		%	56.70%	31.30%	3.70%	1.50%	6.70%	
	Eastern Region	N	120	60	0	6	4	
		%	63.20%	31.60%	0.00%	3.20%	2.10%	
	Southern Region	N	82	41	10	3	3	
		%	59.00%	29.50%	7.20%	2.20%	2.20%	
Education level	None	N	2	0	1	0	0	0.001 *
		%	66.70%	0.00%	33.30%	0.00%	0.00%	
	Up to High School	N	121	47	12	6	0	
		%	65.10%	25.30%	6.50%	3.20%	0.00%	
	Bachelor’s Degree	N	350	175	13	7	16	
		%	62.40%	31.20%	2.30%	1.20%	2.90%	
	Postgraduate and above	N	28	23	2	4	3	
		%	46.70%	38.30%	3.30%	6.70%	5.00%	

* *p* ≤ 0.05 Satistically significant.

**Table 3:** Distribution of perception and knowledge on the use of a toothbrush and tooth paste among Saudi Arabian population.

Question	Answers	Frequency	Percent
1. Do you clean your teeth?	Yes	778	96
	No	32	4
2. What do you use to clean your teeth?	Toothbrush and toothpaste	501	61.9
	Toothbrush, toothpaste, and floss	245	30.2
	Miswak	28	3.5
	I don’t brush my teeth	17	2.1
	Other	19	2.3
3. How often do you clean your teeth?	Twice or once a week	56	6.9
	Not every day	90	11.1
	Once a day	274	33.8
	Twice a day	272	33.6
	More than twice a day	96	11.9
	Not applicable	22	2.7
4. How long do you take to brush your teeth?	About half a minute	181	22.3
	About 1 min	299	36.9
	About 2 min	260	32.1
	More than 5 min	47	5.8
	Not applicable	23	2.8
5. What type of brush do you use?	Hard	52	6.4
	Soft	314	38.8
	Extra soft	41	5.1
	Medium	304	37.5
	Never noticed	77	9.5
	Not applicable	22	2.7
6. What brushing technique do you use?	Horizontal	358	34.1
	Vertical	316	30.1
	Circular	229	21.8
	Toothbrush horizontal and bristles part-way on the gum	111	10.6
	Not applicable	36	3.4
7. How often do you change your brush?	Every 3 months	365	45.1
	Every 6 months	235	29
	When bristles get worn or frayed	166	20.5
	Not applicable	44	5.4
8. What type of toothpaste do you use?	Herbal toothpaste	75	9.3
	Fluoridated toothpaste	266	32.8
	Anti-sensitive toothpaste	180	22.2
	Any toothpaste, whichever is cheaper	32	4
	Toothpaste for whitening teeth	193	23.8
	Not applicable	64	7.9

**Table 4:** Perception and knowledge of different parameters on “The type of brush you use?”.

Variables			Hard	Soft	Extra Soft	Medium	Never Noticed	Not Applicable	*p* Value
Sex	Male	N	29	104	12	96	28	22	0.001 *
		%	10.00%	35.70%	4.10%	33.00%	9.60%	7.60%	
	Female	N	23	210	29	208	49	0	
		%	4.40%	40.50%	5.60%	40.10%	9.40%	0.00%	
Age Group	20–30	N	23	171	28	159	60	8	0.001 *
		%	5.10%	38.10%	6.20%	35.40%	13.40%	1.80%	
	31–40	N	4	57	9	73	11	1	
		%	2.60%	36.80%	5.80%	47.10%	7.10%	0.60%	
	41–50	N	16	55	2	55	4	8	
		%	11.40%	39.30%	1.40%	39.30%	2.90%	5.70%	
	50 and above	N	9	31	2	17	2	5	
		%	13.60%	47.00%	3.00%	25.80%	3.00%	7.60%	
Region	Northern Region	N	11	45	5	50	11	3	0.125
		%	8.80%	36.00%	4.00%	40.00%	8.80%	2.40%	
	Central Region	N	13	82	15	84	24	4	
		%	5.90%	36.90%	6.80%	37.80%	10.80%	1.80%	
	Western Region	N	13	41	6	60	8	6	
		%	9.70%	30.60%	4.50%	44.80%	6.00%	4.50%	
	Eastern Region	N	8	74	10	72	20	6	
		%	4.20%	38.90%	5.30%	37.90%	10.50%	3.20%	
	Southern Region	N	7	72	5	38	14	3	
		%	5.00%	51.80%	3.60%	27.30%	10.10%	2.20%	

* *p* ≤ 0.05 Satistically significant.

**Table 5:** Perception and knowledge of different parameters on “Do you know what interdental aids are?”

1. Do You Know What Interdental Aids Are?					*p* Value
			Yes	No	
Sex	Male	N	220	71	0.001 *
		%	75.60%	24.40%	
	Female	N	460	59	
		%	88.60%	11.40%	
Age Group	20–30	N	382	67	0.139
		%	85.10%	14.90%	
	31–40	N	121	34	
		%	78.10%	21.90%	
	41–50	N	122	18	
		%	87.10%	12.90%	
	50 and above	N	55	11	
		%	83.30%	16.70%	
Region	Northern Region	N	96	29	0.109
		%	76.80%	23.20%	
	Central Region	N	184	38	
		%	82.90%	17.10%	
	Western Region	N	115	19	
		%	85.80%	14.20%	
	Eastern Region	N	167	23	
		%	87.90%	12.10%	
	Southern Region	N	118	21	
		%	84.90%	15.10%	
Education level	None	N	3	0	0.576
		%	100.00%	0.00%	
	Up to High School	N	151	35	
		%	81.20%	18.80%	
	Bachelor’s Degree	N	476	85	
		%	84.80%	15.20%	
	Postgraduate and Above	N	50	10	
		%	83.30%	16.70%	

* *p* ≤ 0.05 Satistically significant.

**Table 6:** Distribution of perception and knowledge on the use of interdental aids and mouthwash among the Saudi Arabian population.

Question	Answers	Frequency	Percent
1. Do you know what interdental aids are?	Yes	680	84
	No	130	16
2. Do you use dental floss or any other device to clean between teeth?	Yes	507	62.6
	No	303	37.4
3. Which of the following interdental aids do you use?	Dental floss	316	39
	Super Floss: A special dental floss that is designated to clean under bridge or between braces	29	3.6
	Interdental brush	43	5.3
	Waterpik flosser	34	4.2
	Toothpick	258	31.9
	Never used any	130	16
4. From the previous question, why did you choose to use these interdental aids?	It is simple and comfortable	352	43.5
	I have space between my teeth and I feel it is more suitable	104	12.8
	I feel it is cleaner	102	12.6
	I have crowded teeth and I feel it is suitable	46	5.7
	It was recommended by my dentist	59	7.3
	Not applicable	147	18.1
5. What type of dental floss do you use?	Waxed	186	23
	Unwaxed	40	4.9
	Never noticed, I use randomly	247	30.5
	I don’t use dental floss	337	41.6
6. Do you use mouthwash or another dental rinse product?	Yes	391	48.3
	No	419	51.7
7. What type of mouthwash do you use?	Fluoride mouthwash	214	26.4
	Natural mouthwash	161	19.9
	Whitening mouthwash	28	3.5
	Not applicable	407	50.2
8. Do you think you can use mouthwash instead of a toothbrush to clean your teeth?	Yes	94	11.6
	No	716	88.4
9. Do you clean your tongue?	Yes	554	68.4
	No	256	31.6
10. What the type of aid do you use to clean your tongue?	With the same toothbrush	396	48.9
	Tongue scrubber/brush	166	20.5
	Not applicable	248	30.6
11. Do you have/wear braces, bridges, or any fixed prosthesis?	Yes	218	26.9
	No	592	73.1
12. Do you use Super Floss or an interdental brush between your teeth or under your bridges?	Yes	203	25.1
	No	171	21.1
	Not applicable	436	53.8
13. When you go to the dentist, did they explain to you how to brush your teeth or how to use interdental aids?	Yes	356	44
	No	454	56

## Data Availability

The data presented in this study are available upon request.

## References

[1] Sharda A., Sharda J. (2010). Factors influencing choice of oral hygiene products used among the population of Udaipur, India. Int. J. Clin. Dent..

[2] Havigerová J.M., Dosedlová J., Burešová I. (2018). One health behavior or many health-related behaviors?. Psychol. Res. Behav. Manag..

[3] Opeodu O.I., Gbadebo S.O. (2017). Factors influencing choice of oral hygiene products by dental patients in a Nigerian teaching hospital. Ann. Ib. Postgrad. Med..

[4] Astrom A.N., Ekback G., Ordell S., Gulcan F. (2018). Changes in oral health-related quality of life (OHRQoL) related to long-term utilisation of dental care among older people. Acta Odontol. Scand..

[5] Laajala A., Pesonen P., Anttonen V., Laitala M.L. (2019). Association of enamel caries lesions with oral hygiene and DMFT among adults. Caries Res..

[6] Moynihan P., Makino Y., Petersen P.E., Ogawa H. (2018). Implications of WHO guideline on sugars for dental health professionals. Commun. Dent. Oral Epidemiol..

[7] Worthington H.V., MacDonald L., Poklepovic Pericic T., Sambunjak D., Johnson T.M., Imai P., Clarkson J.E. (2019). Home use of interdental cleaning devices, in addition to toothbrushing, for preventing and controlling periodontal diseases and dental caries. Cochrane Database Syst. Rev..

[8] Jepsen S., Blanco J., Buchalla W., Carvalho J.C., Dietrich T., Dorfer C., Eaton K.A., Figuero E., Frencken J.E., Graziani F. (2017). Prevention and control of dental caries and periodontal diseases at individual and population level: Consensus report of group 3 of joint EFP/ORCA workshop on the boundaries between caries and periodontal diseases. J. Clin. Periodontol..

[9] Bhaskar N., Gopikrishna V., Kulkarni S., Jacob J., Sourabha K. (2016). Knowledge, attitude, and practices of oral hygiene among college students in Bengaluru city. J. Assoc. Public Health Dent..

[10] Kumar S., Preetha G. (2012). Health promotion: An effective tool for global health. Indian J. Community Med..

[11] Benazir Hussain M., Kalaiselvi Perumal M.P., Santhosh K. (2018). Knowledge, attitude, and practices toward oral hygiene maintenance among patients visiting a dental college. Drug Invent. Today.

[12] Al-Qahtani S.M., Razak P.A., Khan D.A.A.S. (2020). Knowledge and Practice of Preventive Measures for Oral Health Care among Male Intermediate Schoolchildren in Abha, Saudi Arabia. Int. J. Environ. Res. Public Health.

[13] Almulhim B., Alamro B. (2016). Knowledge and attitude toward oral health practice among the parents in Riyadh city. J. Indian Acad. Dent. Spec. Res..

[14] Elsabagh H.M., Abd Elkader M.N., Abd Elkader E.N. (2018). Oral hygiene knowledge, attitude, practice and self-perception of personal dental appearance among majmaah university female student, KSA. Int. J. Adv. Community Med..

[15] Al-Hammadi A.A., Al-Rabai N.A., Togoo R.A., Zakirulla M., Alshahrani I., Alshahrani A. (2018). Knowledge, attitude, and behavior related to use of miswak (Chewing Stick): A cross-sectional study from aseer region, Saudi Arabia. Contemp. Clin. Dent..

[16] Almassri O., Alanazi L., Almutiri J., Asirri S., Sultana F., Alturaif D. (2019). Knowledge, Awareness about Dental Flossing Among Adult Population in Saudi Arabia. Saudi J. Dent. Res..

[17] Abbas Darout I. (2016). Oral Health Related Knowledge and Behavior Among Secondary School Students in Jazan Region, Kingdom of Saudi Arabia. Am. J. Public Health.

[18] Graça S.R., Albuquerque T.S., Luis H.S., Assunço V.A., Malmqvist S., Cuculescu M., Slusanschi O., Johannsen G., Galuscan A., Podariu A.C. (2019). Oral health knowledge, perceptions, and habits of adolescents from Portugal, Romania, and Sweden: A comparative study. J. Int. Soc. Prevent Communit. Dent..

[19] Agrawal A., Gupta A. (2020). Exploring the Factors Influencing the Choice of Oral Care Products: A Review on Personalised Approach. Int. J. Oral Dent. Health.

[20] Kamil M.A., Bashir R.O. (2017). Interdental cleaning aids knowledge, awareness and practices among jazan university students, Saudi Arabia. Int. J. Dent. Health Sci..

[21] Chakraborty M., Thakkar R.R., Swamy D.F., Kumar A., Mehta S., Badiyani B.K. (2017). Knowledge, attitude, and practices about oral hygiene maintenance among patients attending a dental college in India. Int. J. Oral Care Res..

[22] Rajpar S.P., Banglani M.A., Punjabi S.K., Priya M. (2016). Dental floss; concept and use among the undergraduate dental students. Prof. Med. J..

[23] Harini K., Ganapathy D., Visalakshi R.M. (2019). Knowledge, attitude, and awareness on oral hygiene practice among patients wearing fixed partial denture. Drug Invent. Today.

